# Big data integration for enhanced epidemiological research: insights and directions from NHLBI's workshop

**DOI:** 10.3389/fdgth.2026.1770258

**Published:** 2026-04-22

**Authors:** Md Mobashir Hasan Shandhi, Joseph Coresh, Jessilyn Dunn, Eric J. Shiroma, Kenneth J. Wilkins, Dana L. Wolff-Hughes, Ruzhang Zhao, Yuling Hong, Gabriel Anaya

**Affiliations:** 1 School of Electrical, Computer and Energy Engineering; Center for Bioelectronics and Biosensors, Biodesign Institute, Arizona State University, Tempe, AZ, United States; 2 Department Population Health, New York University, Grossman School of Medicine, New York, NY, United States; 3 Department of Medicine, New York University, Grossman School of Medicine, New York, NY, United States; 4 Department of Biomedical Engineering, Duke University, Durham, NC, United States; 5 Department of Biostatistics and Bioinformatics, Duke University, Durham, NC, United States; 6 Division of Cardiovascular Sciences, Prevention and Population Sciences Program, Clinical Applications and Prevention Branch, National Institutes of Health, National Heart, Lung, and Blood Institute, Bethesda, MD, United States; 7 National Institutes of Health, National Institute of Diabetes and Digestive and Kidney Diseases, Office of the Director, Bethesda, MD, United States; 8 Division of Cancer Control and Population Sciences, National Institutes of Health, National Cancer Institute, Bethesda, MD, United States; 9 Department of Biostatistics, Johns Hopkins University, Baltimore, MD, United States; 10 Division of Cardiovascular Sciences, Prevention and Population Sciences Program, Epidemiology Branch, National Institutes of Health, National Heart, Lung and Blood Institute, Bethesda, MD, United States

**Keywords:** artificial intelligence in healthcare, big data integration, digital biomarkers, digital epidemiology, life-course data linkage, wearable devices

## Abstract

The landscape of epidemiological research is experiencing a technological transformation, driven by the rapid expansion of big data and advancements in artificial intelligence (AI) and machine learning (ML). This workshop explored the opportunities and challenges associated with integrating diverse data sources into population-based research at different levels, including electronic health records (EHRs), genomic and omics data, imaging, wearable device data, and social determinants of health measures, among others. AI/ML tools present powerful capabilities for analyzing these vast datasets, offering advancements in health risk prediction, disease pattern identification, and the development of personalized interventions. However, the integration of big data introduces technical barriers related to data heterogeneity, privacy and security concerns, and the potential to exacerbate health disparities through algorithmic biases. In September 2023, the National Institutes of Health's (NIH) National Heart, Lung, and Blood Institute (NHLBI), in collaboration with the National Cancer Institute (NCI) and the National Institute of Diabetes and Digestive and Kidney Diseases (NIDDK), hosted a workshop to address these challenges and discuss the integration of big data into epidemiology and population-based studies. Key themes from the workshop emphasized interdisciplinary collaboration, data standardization, and the development of robust ethical frameworks, as well as the importance of advancing data governance, implementing transparent consent processes, and employing privacy-preserving techniques to maintain public trust. Additionally, the workshop highlighted the transformative potential of digital health technologies, such as wearable devices, which, when integrated with EHRs, enhance data granularity, facilitate early disease detection, and strengthen public health surveillance. Ethical, legal, and social issues (ELSI) are central to responsibly leveraging big data and AI in research, unbiased algorithms, the use of diverse datasets in AI training, and continuous human oversight to mitigate risk and ensure validity. The workshop also emphasized the need for workforce training and education in data science and bioinformatics to prepare researchers for utilizing these technologies effectively. The workshop concluded by recognizing the need for a balanced approach that addresses data integration challenges while harnessing AI/ML to improve healthcare outcomes. By fostering interdisciplinary collaboration, prioritizing privacy, and embracing data-driven methodologies, epidemiological research can unlock the full potential of big data to transform public health and clinical practice.

## Introduction

The landscape of epidemiological research is undergoing a transformative shift, driven by the exponential growth of data and the rapid advancement of artificial intelligence (AI) tools and machine learning (ML). This abundance of “big data”, encompassing electronic health records (EHR), -omics, imaging, wearable devices, and social determinants of health measures, and many other data types, presents an unprecedented opportunity to understand the complexities of human health and disease. Simultaneously, AI/ML tools, with their capacity for pattern recognition and predictive analytics, offer powerful methods for analyzing these massive datasets, extracting meaningful insights, and translating them into improved health outcomes.

However, this shift also brings significant challenges. Integrating data from disparate sources with varying formats and quality presents technical hurdles. Ethical considerations regarding data privacy, security, and potential re-identification of participants necessitate robust safeguards. Additionally, biases inherent in data collection and algorithm design can exacerbate health disparities, highlighting the need for unbiased approaches and transparency in methodology.

Recognizing both the immense potential and the complexities of this evolving landscape, in September 2023 the NIH hosted a collaborative workshop, “*Big Data Integration: Unlocking the Potential for Enhanced Epidemiological Research*”. This partnership between NHLBI, NCI, and NIDDK, brought together leading experts to explore the complexities and opportunities associated with harnessing the potential of big data in epidemiology and population-based research.

By its conclusion, the workshop had outlined a forward-looking agenda for population-based research in the digital age. The collective insights and strategies discussed pointed towards a future where big data and AI/ML enhance our understanding of health and disease across the lifespan, as well as drive impactful innovations in public health policy and clinical practice. This vision emphasized the critical role of interdisciplinary collaboration, data standardization, and innovative analytical methodologies in unlocking the transformative potential of epidemiological research powered by big data.

## Workshop objectives

The workshop was designed to address key objectives to advance the field of epidemiology through the lens of big data. These objectives addressed a wide range of topics, including enhancing the understanding of big data's role in population-based research, emphasizing the importance of data standards and harmonization to ensure research reproducibility, and promoting innovative strategies for data integration.

Specifically, the workshop sought to assess the current landscape of big data in biomedical research, identifying existing gaps and opportunities for the optimal use of these resources in analyzing population health. A major focus was placed on recognizing the opportunities for a harmonized ecosystem that facilitates the seamless integration of diverse data sources.

The workshop discussed and reviewed existing methodologies alongside emerging data science strategies, with a need for standardized data practices to improve the robustness and reproducibility of research findings. Furthermore, the workshop actively pursued discussions on effective data integration techniques, leveraging established tools and methodologies to advance population-based research.

Additionally, the workshop explored strategies for efficiently combining various types of data, applying novel data science tools, and fostering an environment that encourages collaborative and interdisciplinary research. By promoting knowledge-sharing and addressing challenges collectively, the workshop highlighted the importance of data diversity, data integration, and the use of advanced methodologies to drive innovation in epidemiology.

## Digital health landscape

The field of epidemiology is undergoing a transformative revolution fueled by the expanding options for measurement of digital biomarkers derived from wearables and smart devices (1), offering non-invasive and remote means to monitor various physiological systems. These biomarkers, which include parameters like location and heart rate, provide valuable insights into an individual and population-level health status (2, 3).

With rapid innovations in hardware and computational algorithms, digital health technologies (DHTs) are revolutionizing health monitoring, with widespread adoption among the general population—85% of Americans own smartphones (4) and 31% own smartwatches (5). Successful applications in epidemiological and clinical research—if not basic experimental studies involving humans (BESH) designs or device studies (6)—include irregular heart rhythm monitoring (7), sleep health assessment (8), physical activity monitoring (9), food logging (10), and mental stress monitoring (11), among others. Innovations like sensor-embedded fabrics, banded electronics, and tattoo electronics are emerging, ensuring seamless integration into daily life. For instance, temporary tattoos can discreetly monitor heart rhythms (12), smart rings can track sleep health and vital signs (13), and smart clothes can monitor physiological signals.

Wearable devices for continuous health monitoring represent a significant advancement in early detection of health issues, even before symptoms appear (14). Integrating wearables with healthcare systems improves the accuracy and timeliness of health data, facilitating early detection and intervention. Wearables provide objective, quantitative measurements, reducing reliance on potentially biased self-reported data and filling gaps between clinical visits (15). This real-time monitoring allows for timely alerts and early intervention, potentially preventing disease progression or complications. Continuous monitoring is especially valuable for managing chronic diseases, allowing for precise adjustments to treatments and improving patient outcomes and healthcare efficiency. The potential cost savings from early disease detection through wearable technology could be substantial.

Integrating wearable devices sensor data with EHR enhances data granularity and completeness in epidemiological and clinical research, subject to measurement limitations empirically assessed within BESH designs. Wearable devices sensors provide continuous, real-time monitoring of physiological parameters, complementing periodic EHR entries and creating a comprehensive health picture over time (16). This high-resolution data captures short-term variations and trends, leading to more robust datasets for research.

From a broader perspective, integrating wearable data with EHR enhances public health monitoring and research capabilities. Aggregated data from wearables can reveal public health trends, monitor disease outbreaks, and assess intervention effectiveness (17). Wearables can also capture contextual information, such as location, to study environmental impacts on health. This integration supports personalized medicine by providing insights into individual health patterns and behaviors, enabling tailored interventions. Overall, integrating wearable device sensor data with EHR leads to more effective healthcare delivery, cost savings, and improved health outcomes through advanced analytics and real-time monitoring.

DHTs can significantly enhance patient outcomes by improving healthcare delivery and fostering patient engagement. Tools like telemedicine, mobile health apps, and wearable devices increase accessibility and convenience, allowing remote consultations and improving access to care, especially in underserved areas. Mobile health apps facilitate self-monitoring and management of chronic diseases, while wearable devices enable real-time health tracking and early detection of potential issues. These technologies contribute to timely and accurate diagnoses, better chronic condition management, and overall improved patient outcomes. DHTs empower patients by providing easy access to health information and educational resources, encouraging active health management and better adherence to treatments.

On a broader scale, digital health positively impacts public health by enabling advanced data analytics and improving population health management. The vast amounts of data generated by digital health tools can be analyzed to identify trends, predict outbreaks, and evaluate public health interventions. Health authorities can use DHT data to make informed decisions, allocate resources effectively, and design targeted health programs (18). Digital health platforms can facilitate large-scale public health campaigns and provide real-time information during health emergencies, enhancing disease surveillance and response capabilities (19). By leveraging data and technology, public health officials can improve health outcomes on a population level, ensuring a more efficient, accessible, and effective healthcare system.

Despite the potential of wearables and digital biomarkers in epidemiology, challenges persist in their discovery and utilization (16). Ensuring the validity and accuracy of data, interpretability, and adherence to FAIR principles (making data Findable, Accessible, Interoperable, and Reusable) are paramount. Regulatory oversight is necessary, especially as many devices may be geared toward consumer use rather than designed for rigorous data collection for epidemiological or clinical purposes. Additionally, the sheer volume of data generated by these devices, coupled with inherent noise, poses challenges in data management and analysis.

Besides, selection bias is a big concern with wearables and digital health technologies. Electronic health records reflect patterns of healthcare utilization rather than true population-wide disease burden. Similarly, wearable and bring-your-own-device (BYOD) study designs may overrepresent individuals with higher socioeconomic status, digital literacy, or health engagement. These participation patterns can introduce systematic bias and limit generalizability.

Additional practical limitations warrant explicit consideration. Many commercial wearable devices rely on proprietary algorithms with limited access to raw sensor signals, constraining reproducibility and independent validation. Firmware and software updates may alter signal processing pipelines, leading to temporal instability in derived digital biomarkers. Cross-device variability and limited harmonization standards complicate multi-cohort integration efforts. Besides, performance differences across skin tone, age, body mass index, and comorbid conditions have been documented, raising important equity concerns. The rapidly evolving hardware ecosystem also introduces reproducibility challenges for long-term epidemiologic studies, where device generations may change during follow-up. These challenges can alter measurement characteristics over time, complicating longitudinal inference and cross-cohort harmonization. Missing-not-at-random mechanisms are common in longitudinal digital datasets. Device non-wear, intermittent syncing, data dropout, and healthcare disengagement may correlate with health status, leading to biased estimates if not explicitly modeled.

Addressing these challenges necessitates the development and utilization of appropriate tools and frameworks. Ethical and legal issues must be carefully considered, particularly regarding privacy, security, and the balance between risks and benefits for participants (20). Robust data governance mechanisms and transparent consent processes are essential to safeguard individuals' rights and interests. Examples of such mechanisms may include data access committees with appropriate scientific and community representation, dynamic consent models that allow participants ongoing control over data use, and documentation and audit structures that support transparency, accountability, and evaluation of AI systems in population research. Furthermore, interdisciplinary collaborations involving epidemiologists, data scientists, clinicians, ethicists, patient advocacy groups, professional societies, and policymakers are essential to navigate the complex landscape of digital epidemiology responsibly and ethically. By leveraging real-world data from wearables and smart devices in conjunction with comprehensive frameworks addressing these concerns, next-generation epidemiology holds immense promise in transforming our understanding and management of public health.

## Data integration and the role of AI in epidemiological research

The future of epidemiological research depends on the effective integration of diverse data sources, offering a comprehensive and dynamic picture of population health. This integration involves harmonizing data from disparate sources, such as EHRs spanning an individual's life, a variety of wearable devices with proprietary software, large-scale biomedical data repositories (biobanks) containing genomic and -omics data, as well as other data sources such as images, videos, social media, environmental measures, social determinants of health (SDOH) measures, and more. Navigating this landscape requires addressing technical challenges and ethical considerations (21–23).

One key challenge is data heterogeneity. Healthcare data often vary in format and quality due to inconsistent coding practices across healthcare systems (24). Similarly, wearable devices data, despite offering real-time insights, differ in interpretability based on device brands, standards, and limited access to raw data (25–27). While national standardization efforts are being adopted—such as the Assistant Secretary for Technology Policy (ASTP)/Office of the National Coordinator's (ONC) United States Core Data for Interoperability (USCDI), additional efforts by Standards Development Organizations (e.g., IEEE 1752, ANSI/CTA 2065), and frameworks like Health Level 7's (HL7) Fast Healthcare Interoperability Resources (FHIR), NIH-endorsed common data elements, and common data models (OMOP, PCORnet, i2b2, Sentinel, TriNetX, among other s^28^, ^29^) are advancing. Although achieving seamless interoperability remains complex (30, 31), enhancing FAIR data frameworks support the creation of reproducible research environments (32).

Even within standardized formats, data variability persists. Studies, such as those using the UK Biobank, illustrate this: when integrating risk factors with proteomics data, less than 10% of the cohort has complete data sets, complicating comprehensive analyses (33, 34). Moreover, ensuring data privacy and confidentiality remains central, advanced techniques like differential privacy and federated computation show promise in protecting participant data while maintaining research utility (35–38). Beyond technical solutions, fostering trust and transparency with research participants is vital. Initiatives like the *All of Us* Research Program demonstrate a participant-centric approach that prioritizes returning value to individuals and fostering engagement (22, 39).

Biases in data collection and analysis are another concern, particularly regarding generalizability of findings. Algorithmic bias can arise from training and validating models on non-representative data, leading to inaccurate predictions and exacerbating disparities (40). To address this, researchers must adopt bias balancing strategies, incorporating diverse datasets and considering social determinants or drivers of health (23, 24, 41). In addition, underreporting of certain demographic factors (e.g., race and ethnicity) in datasets can have downstream effects in algorithm development due to embedded biases with non-representative data (42), even when addressed using lessons learned for specific sources (e.g., EHR s^43^). To tackle the lack of representation in demographic reporting from biosignal/biomarker databases, standardized guidelines that include detailed, participant-level demographic data could be established. These guidelines could be based on the Office of Management and Budget's Statistical Policy Directive No. 15 (44), which sets standards for maintaining, collecting, and presenting federal data on race and ethnicity, or on the NIH's policy and guidelines for reporting demographics in human participant studies, which mandate the reporting of race, ethnicity, and sex at the participant level (45).

Successful data integration efforts, such as the Cohorts for Heart and Aging Research in Genomic Epidemiology (CHARGE) Consortium and the Atherosclerosis Risk in Communities (ARIC) study, can show the value of collaboration and long-term data collection. The CHARGE Consortium's federated model has driven significant genomic discoveries, while the ARIC study's longitudinal data have illuminated chronic disease trajectories (46, 47). The impact of such integration extends beyond research, influencing public health policies, informing resource allocation, and guiding targeted interventions to reduce health disparities (30, 48, 49).

AI is enhancing epidemiological research by providing scientists with sophisticated tools to analyze complex and large volumes of data, identify novel insights, and predict health outcomes with unprecedented accuracy. This application of AI is already improving predictive modeling, medical imaging, diagnostics, natural language processing, and surveillance, among other areas of medicine and population research (49–51). AI can also sift through vast health datasets, forecasting individual risks for chronic diseases such as cardiovascular disease, diabetes, dementia, cancer, and other chronic diseases and improving clinical trials on these conditions (52). Some of these models can integrate genetic, lifestyle, and environmental data and enable tailored prevention strategies and early interventions (53–56).

Despite its promise, AI introduces privacy concerns when using personal health information, necessitating robust safeguards and privacy-preserving technologies (57–59). The “black-box” nature of many AI models raises questions about transparency and accountability, emphasizing the need for explainable AI that healthcare professionals and patients can trust (60–63). Moving forward, incentivizing data sharing while safeguarding privacy is needed. Developing secure computational infrastructure and standardized data protocols will facilitate integration efforts. Training future researchers in data science and bioinformatics will also be essential (64, 65). Addressing these challenges requires designing unbiased models, incorporating human oversight, and establishing comprehensive ethical frameworks (66, 67) that should prioritize privacy, transparency, fairness, and accountability, ensuring AI is harnessed to advance public health.

## Data integration across the lifespan

**Figure 1:**
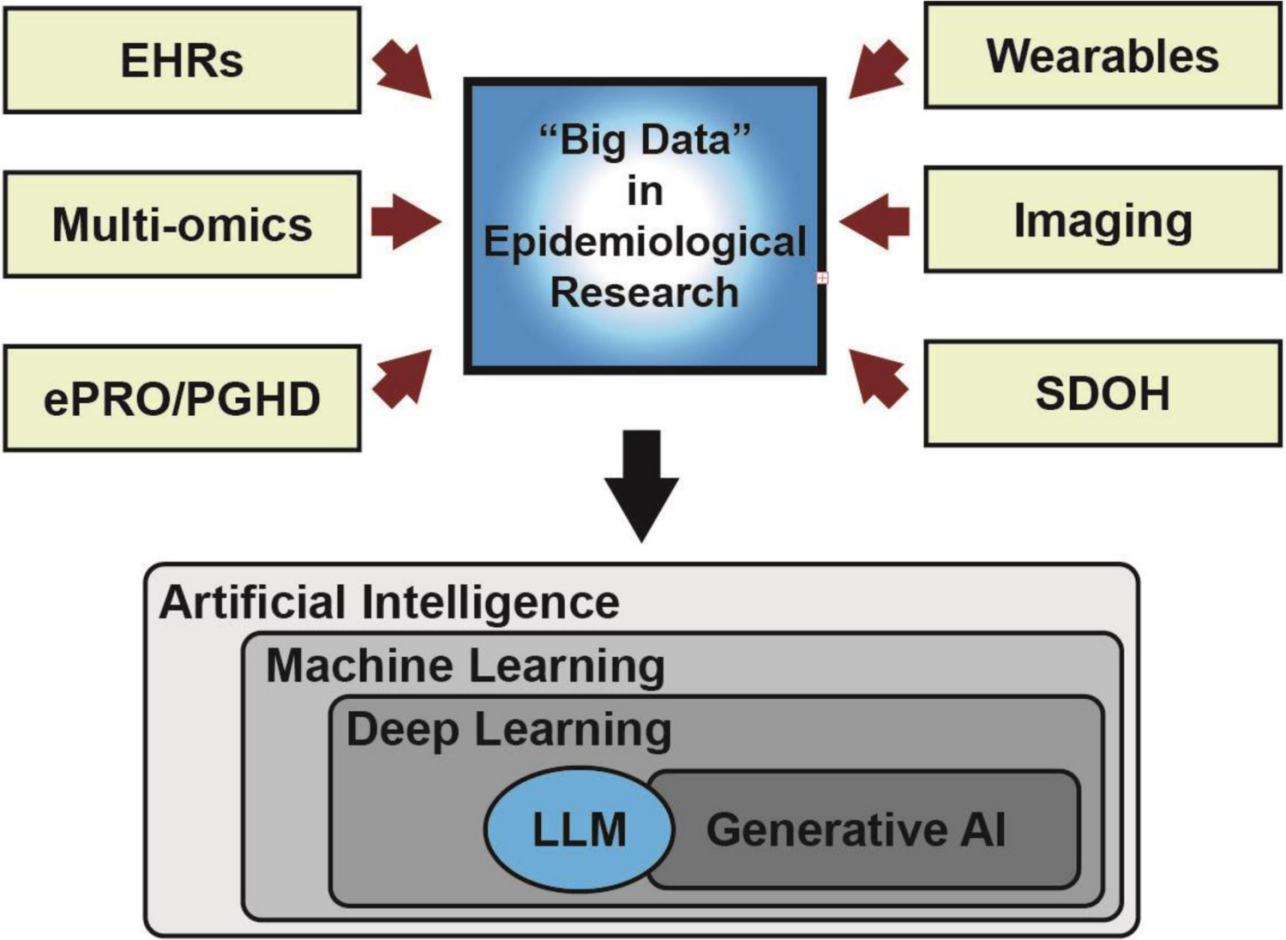
Framework for integrating big data and artificial intelligence in epidemiological research. This figure illustrates the conceptual framework for leveraging big data and AI/ML in epidemiology. It highlights the diverse data sources, including electronic health records, genomic and omics data, imaging, wearable devices sensor data, and social determinants of health.

Integrating data across the life span is a transformative approach in research, particularly in population-based studies (Figure 1). This method offers a comprehensive understanding of health trajectories from prenatal stages through older age. This approach leverages the interconnectedness of modern data ecosystems, linking diverse data sources to yield insights into health determinants, enable proactive epidemiological monitoring, and drive personalized interventions. At the same time, cross-system linkage requires careful methodological consideration, as probabilistic matching approaches, duplicate records, and identity resolution processes may introduce linkage error or misclassification that should be considered for in life-course analyses.

The importance of life course data integration lies in its ability to provide a holistic view of an individual's health journey from “cradle to grave” and allowing for the identification of critical periods where interventions can have the most significant impact. For instance, early-life exposures to environmental toxins (68, 69), poor nutrition, or lifestyle behaviors can have long-term effects on health (70). Longitudinal health records, spanning childhood, adolescence, and adulthood, capture a wide range of information, from vaccination histories and physical fitness assessments to chronic disease diagnoses and lifestyle factors (71, 72).

Advances in big data analytics, including AI, have further enhanced the utility of life course data integration (Figure 1) (73). These tools can identify patterns and relationships that may not be apparent through traditional statistical methods, enabling the prediction of individual health risks and the identification of intervention points (49, 50, 74). By continuously analyzing health data across the lifespan, these models can support the development of personalized prevention strategies and early interventions, shifting the focus from reactive to proactive healthcare (53, 75, 76).

Data integration also enhances public health surveillance and response. This capability has been timely for public health interventions and managing communicable diseases. For instance, during the COVID-19 pandemic, AI tools were employed to track the spread of the virus, predict hotspots, and notify about possible exposure, demonstrating the potential of integrated data in public health (77–79). While these benefits are substantial, it also presents ethical and privacy challenges. Protecting personal health information requires stringent privacy measures, like encryption and anonymization, while algorithmic biases must be identified and mitigated. Ensuring fairness in data analysis involves designing inclusive models trained on diverse datasets to avoid reinforcing existing disparities.

## Gaps and opportunities

**Figure 2:**
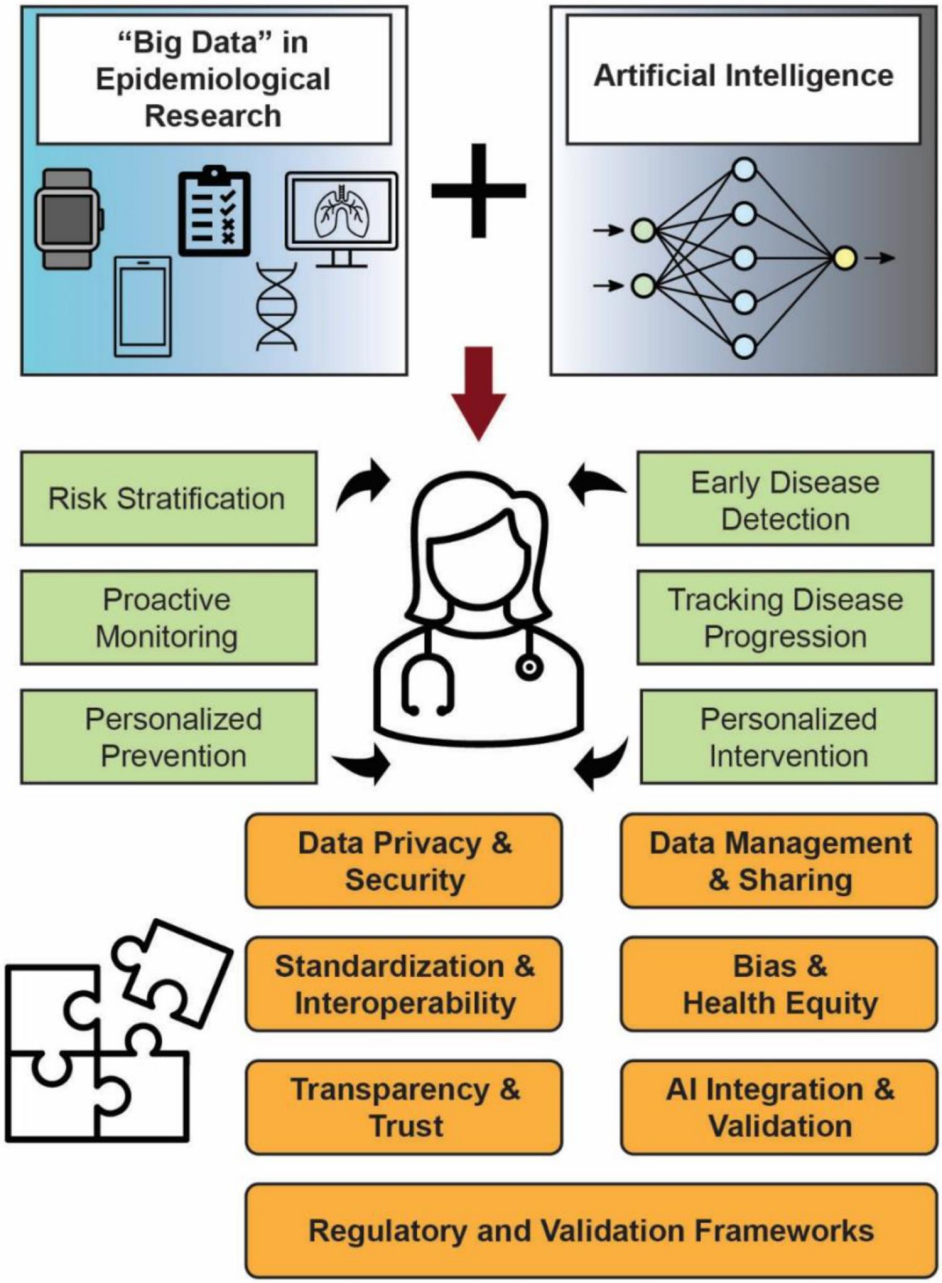
Identifying gaps and opportunities in big data and AI-driven epidemiological research. This figure outlines the key gaps and opportunities in the integration of big data and AI within epidemiological research. It emphasizes the need for interdisciplinary collaboration, data standardization, and robust ethical frameworks to address technical barriers and privacy concerns. These gaps are structured as example actionable domains requiring coordinated methodological, infrastructural, ethical, and policy investments.

The integration of digital health tools, life course data, and the application of AI/ML in epidemiological and population-based research offers transformative potential but also highlights several gaps that need to be addressed (Figure 2). These gaps and opportunities emphasize the importance of strategic direction for leadership, the research community, and the general public.

- **Data Privacy and Security:** Ensuring data privacy and security while promoting data sharing remains a challenge. The risk of re-identification and data breaches can deter data sharing, necessitating the development of robust computational infrastructures that provide the necessary security. Streamlining standardized protocols for data collection and reporting, along with implementing advanced privacy-preserving techniques, can enhance data protection and foster a culture of data sharing.
- **Data Management and Sharing:** Effective management of longitudinal data, refining algorithms for large-scale datasets, and establishing efficient data-sharing networks are needed. Addressing barriers to the adoption of standards and exploring economic models for sustainable interoperability will facilitate collaboration. Shared digital infrastructures that support data harmonization and real-time analysis are needed to advance population health research.
- **Standardization and Interoperability:** Variability in data formats and coding practices across healthcare systems complicates data integration. Efforts to promote interoperability and adhere to ONC's USCDI guidelines are encouraged. Addressing these gaps requires collaboration among standards bodies, researchers, and healthcare organizations to create universally accepted data models and improve data quality across platforms.
- **Data Quality:** Biases inherent in data collection and algorithm design can exacerbate existing health disparities. It is important to train AI models on diverse and representative datasets, design algorithms that account for limitations in the data, and maintain continuous human oversight.
- **Ethical, Legal, and Social Implications:** Addressing concerns related to data privacy, security, risks-benefits, informed consent, and access for participants is still necessary. Concrete governance approaches, like structured data access review processes with appropriate scientific and community representation, participant-centered or dynamic consent models, transparent documentation and audit mechanisms for AI systems, and processes for ongoing evaluation of model performance and equity across populations, may help operationalize these principles in practice. Embracing ethical considerations and fostering interdisciplinary collaborations involving epidemiologists, data scientists, clinicians, ethicists, patient advocacy groups, professional society officials, and policymakers will be key to navigating these challenges.
- **Transparency and Trust:** AI decision-making processes are often opaque, making it difficult for clinicians and patients to understand and trust AI-driven insights. Research should prioritize explainability and clearly communicate the limitations of AI models.
- **Synthetic Data and Privacy:** Synthetic data offers a promising solution for balancing data utility and privacy. Research is needed to evaluate its effectiveness and establish standards for its use. Exploring synthetic data's role in education and workforce development, as well as its potential to simulate health conditions, could provide valuable insights while minimizing risks to patient privacy.
- **AI Integration and Validation:** AI tools need rigorous lifecycle evaluation extending beyond initial model development. Developing AI models that perform well across diverse populations and clinical settings is essential for widespread adoption. Internal validation alone is insufficient. External validation across independent datasets is critical to assess transportability. Integrating AI into clinical workflows requires careful consideration of usability and the impact on healthcare delivery, along with ongoing performance monitoring. Dataset shift, where model performance degrades due to changes in population characteristics, care practices, device ecosystems, or coding standards, must be anticipated and monitored. Model calibration should be routinely assessed to ensure predicted probabilities reflect observed risk. Prospective and pragmatic trials are necessary to evaluate clinical utility, not merely predictive accuracy. Post-deployment surveillance is required to detect model decay over time and unintended disparities in performance. Embedding continuous performance auditing and updating mechanisms within governance frameworks is essential for safe and equitable AI deployment in population health research.
- **Regulatory and Validation Frameworks for Digital Health:** As DHTs such as wearable devices and digital biomarkers become more prevalent, establishing clear regulatory and validation frameworks is essential. Ensuring these tools meet rigorous scientific and clinical standards will support their integration into research and healthcare settings. Collaboration with regulatory agencies to streamline approval processes for innovative technologies will accelerate their impact.
- **Workforce Training and Education:** There is a notable shortage of researchers trained in data science and biomedical informatics, which hampers the effective utilization of big data and AI/ML tools in epidemiology. Investing in educational initiatives to train future generations of researchers in these fields is crucial. Encouraging interdisciplinary education that combines epidemiology with advanced data science techniques will empower researchers to leverage these technologies effectively.

## Conclusion

Epidemiological and population-based research is undergoing a technological transformation driven by big data and advancements in AI and ML. These innovations offer a unique opportunity to better understand the complexities of human health and disease, paving the way for more effective and proactive approaches to healthcare. However, fully harnessing this potential requires addressing challenges of data heterogeneity, privacy and ethical concerns.

Data integration across the life span provides a comprehensive understanding of how health evolves over time. By linking data from various sources, such as EHR, wearable devices, genetic databases, and social determinants of health, while recognizing and addressing potential linkage error and data fragmentation, researchers can identify interventions where preventive measures can make the biggest difference.

Today, the variability and inconsistency in data formats present hurdles that must be overcome through standardization efforts. Enhancing data harmonization and adhering to FAIR principles are essential for creating robust, interoperable datasets that drive meaningful research.

AI and ML offer powerful tools to analyze vast, complex datasets, uncover hidden patterns, predict individual health risks. However, improved prediction may not inherently translate into improved health outcomes. Translation into clinical or public health impact requires carefully designed intervention studies, implementation strategies, and ongoing evaluation of real-world effectiveness. Nonetheless, these technologies must be implemented with caution. Addressing issues of algorithmic bias, transparency, data privacy, dataset shift, model decay over time, calibration, and transportability is crucial to ensure that AI applications are both fair and reliable. Emphasizing explainable models and maintaining human oversight are key to fostering trust in these systems.

While predictive analytics has driven much of the enthusiasm around AI in epidemiology, it is essential to distinguish prediction from causal inference. Accurate prediction of risk does not necessarily imply that modifying the predicted factor will improve outcomes. Improved prediction alone may not guarantee clinical or public health benefit without intervention studies and implementation frameworks. Life-course integration further introduces time-varying confounding, where prior health states influence both future exposures and outcomes. Addressing these complexities requires robust epidemiologic designs, including causal modeling frameworks, sensitivity analyses, calibration strategies, transparent reporting of assumptions, and careful distinction between predictive and causal objectives.

Wearable technology has emerged as a game-changer in continuous health monitoring. When integrated with traditional health records, wearables can fill data gaps, facilitate early diagnosis, and enhance chronic disease management. Yet, challenges in validating digital biomarkers and managing large data volumes remain.

Ultimately, realizing the full benefits of big data and AI in epidemiology requires a collaborative, interdisciplinary approach. This includes investing in education and training programs to equip researchers with data science and biomedical informatics skills and fostering partnerships between epidemiologists, data scientists, clinicians, ethicists, patient advocacy groups, professional society officials, and policymakers. The future of epidemiological research is promising, characterized by advanced technologies and diverse data that enable a more proactive, data-driven healthcare landscape.

## Data Availability

The original contributions presented in the study are included in the article/Supplementary Material, further inquiries can be directed to the corresponding authors.
